# A Virtual Microscope for Academic Medical Education: The Pate Project

**DOI:** 10.2196/ijmr.3495

**Published:** 2015-05-11

**Authors:** Christoph Brochhausen, Hinrich B Winther, Christian Hundt, Volker H Schmitt, Elmar Schömer, C James Kirkpatrick

**Affiliations:** ^1^Laboratory for Regenerative Pathology and Interface Research (REPAIR-Lab)Institute of PathologyUniversity Medical CentreMainzGermany; ^2^Computational Geometry/Computer GraphicsInstitute of Computer ScienceJohannes Gutenberg UniversityMainzGermany

**Keywords:** whole-slide imaging, WSI, virtual microscopy, telepathology, e-learning, databases, Internet, microscopy

## Abstract

**Background:**

Whole-slide imaging (WSI) has become more prominent and continues to gain in importance in student teaching. Applications with different scope have been developed. Many of these applications have either technical or design shortcomings.

**Objective:**

To design a survey to determine student expectations of WSI applications for teaching histological and pathological diagnosis. To develop a new WSI application based on the findings of the survey.

**Methods:**

A total of 216 students were questioned about their experiences and expectations of WSI applications, as well as favorable and undesired features. The survey included 14 multiple choice and two essay questions. Based on the survey, we developed a new WSI application called Pate utilizing open source technologies.

**Results:**

The survey sample included 216 students—62.0% (134) women and 36.1% (78) men. Out of 216 students, 4 (1.9%) did not disclose their gender. The best-known preexisting WSI applications included Mainzer Histo Maps (199/216, 92.1%), Histoweb Tübingen (16/216, 7.4%), and Histonet Ulm (8/216, 3.7%). Desired features for the students were latitude in the slides (190/216, 88.0%), histological (191/216, 88.4%) and pathological (186/216, 86.1%) annotations, points of interest (181/216, 83.8%), background information (146/216, 67.6%), and auxiliary informational texts (113/216, 52.3%). By contrast, a discussion forum was far less important (9/216, 4.2%) for the students.

**Conclusions:**

The survey revealed that the students appreciate a rich feature set, including WSI functionality, points of interest, auxiliary informational texts, and annotations. The development of Pate was significantly influenced by the findings of the survey. Although Pate currently has some issues with the Zoomify file format, it could be shown that Web technologies are capable of providing a high-performance WSI experience, as well as a rich feature set.

## Introduction

### Background

Whole-slide imaging (WSI), also known as virtual microscopy, has become more and more important in e-learning during the past decade. It is being used for general educational purposes, graduate education, pathology training, tutoring, and virtual workshops [1]. In many settings, WSI has already replaced the conventional microscope [1]. Recognizing the potential benefits of such applications for medical education, we conducted a survey to determine students’ expectations of WSI applications for teaching purposes. Based on these findings, a WSI application for pathological specimens—the Pate application [2]—has been developed. The primary goal of developing Pate was to provide a tool that enables students to improve their skills in identifying histomorphological and pathological features on virtualized histological slides. Furthermore, Pate offers the possibility to explain the pathogenesis and pathophysiology of diseases on the basis of the morphological correlation.

### Preexisting Applications

There already exist several WSI applications for histological and histopathological training, mainly the following: (1) Histologiekurs [3] from Zurich University, Switzerland, (2) Mainzer Histo Maps [4] from the Johannes Gutenberg University Mainz, Germany, (3) NYU Virtual Microscope (NYUVM) [5] from New York University, USA, (4) ScanScope Images [6] from Zurich University, Switzerland, (5) Virtuelle Pathologie Magdeburg [7] from the Otto von Guericke University, Magdeburg, Germany, (6) vMic [8] from Basel University, Switzerland, and (7) VSlides (Pathorama) [9] from Basel University, Switzerland.

As a basic characteristic of WSI, all these applications support latitude in the slide with a varying degree of usability. Some of the applications, such as ScanScope Images, Histologiekurs, and Mainzer Histo Maps, support annotations. Furthermore, Histologiekurs provides background information about the donor, informational texts about the specimen as well as support for points of interest. However, none of the listed applications, except NYUVM, support small-screen devices or a touch interface. Several of these WSI applications use Adobe Flash to implement the client. This requires the user to install a browser plug-in before using the application. Moreover, many recent devices, such as any Apple mobile gear, do not support those plug-ins. WSI applications using proprietary plug-ins include vMic, Mainzer Histo Maps, ScanScope Images, and VSlides. In contrast, there are also WSI applications that use Hypertext Markup Language 5 (HTML5) technologies. These include the applications NYU Virtual Microscope and Virtuelle Pathologie Magdeburg. By using HTML5 technologies, these applications avoid the disadvantages of browser plug-ins.

Most applications—Flash, Silverlight, and HTML5 based alike—miss important features, such as points of interest (POI), advanced annotations, informational texts, or a map scale. They provide basic image presentation capabilities, but fail to support important features provided by the WSI technology.

### Demand Assessment

Due to the variety of options offered by new WSI technologies [10-12], it was a major prerequisite for a new WSI tool to investigate which features would benefit students, without compromising the usability of Pate. Therefore, the users’ opinions were crucial in identifying and eliminating undesirable features. To achieve this goal, a survey was conducted targeted to functional needs and usability from students’ perspectives as potential users of a new WSI tool. Therefore, their expectations of Pate, their experiences with already existing WSI tools, positive and negative features, as well as feature suggestions were analyzed by a questionnaire.

### Slide Acquisition

As stated by Glatz-Krieger et al [8], the quality of virtual slides is defined by four crucial parameters, namely, the quality of the histological section, the completeness of the histological section, the quality of the scanned image, and the usability of the virtual slides. From these parameters, the quality and completeness of the section should be guaranteed during the physical slide acquisition. These parameters are essential for high-quality slide imaging.

### Image Quality

The image quality is highly influenced by optical focusing during slide scanning. For this, two main methods are currently available. The first method utilizes stacking of multiple planes with different focus settings—z-stacking [13]—which emulates a physical microscope more closely [14]. This method also leads to more memory consumption. However, the slide acquisition process is less complicated, since only the middle optical plane needs to be positioned near the mean focal plane of the glass slide.

The second method uses a single virtual focal plane that resembles the best focus throughout the whole glass slide. Because this procedure results in smaller memory consumption, it was chosen for digitizing the histopathological slides for Pate. In order to ensure optimal results, we manually inspected the suggested automatically generated focal points of the software and corrected them where necessary.

### Usability

Features for optimal usability are smoothly scrolling images, short access times, orientation, and several options for magnification. Furthermore, a good user interface design is of major importance. To achieve this, we put special emphasis on mobile devices next to the classical desktop. Therefore, we wanted to support small screens as well as a touch interface.

### Resources

The Pate suite offers a family of differently scaled versions for each of the high-resolution images [15]. Thus, a user can conveniently choose the scale of interest while having fast response times due to the small bandwidth used during data transfer. This multiresolution representation can be obtained by a cascade of downsampling operations on a dyadic grid similar to the discrete Haar-wavelet decomposition of rasterized images [16]. At first sight, providing a family of images for different resolutions appears inefficient in terms of memory usage. As we will show, the storage size of the multiresolution representation of images used with tiles is bounded by merely 133% of the original image size.

Every downsampling operation halves the length of the given images for each of the two dimensions. Thus, the overall number of pixels is quartered every iteration. Consider the sequence, (*s*_*i*_)_*i*_, of relative sizes according to the original image size, *S*:

(*s*_*i*_)_*i*_ = (1,1/4,1/16,1/64,...)=(*q* ^0^,*q* ^1^,*q* ^2^,*q* ^3^,...), where *q*=1/4.

**Figure 1:**
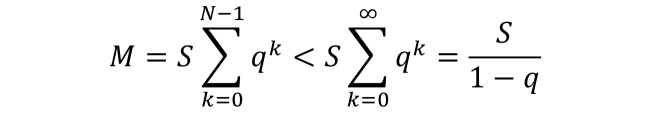
The overall size M can be written in terms of a finite geometric series, where N is the number of downsampling operations.

As a result, the overall size, *M*, can be written in terms of a finite geometric series, as displayed in Figure 1.

The expression for *M* is overestimated by the (infinite) geometric series for *N*→ ∞ which converges for all *q* satisfying 0≤| *q* |<1 [17]. For the choice, *q*=1/4, the overall memory consumption relative to the original image size is obtained as *M*/*S*<4/3. Thus, all resolutions can be stored with less than one-third of additional memory.

## Methods

### Student Survey for the Expectations of a Virtual Microscope

The student survey was designed in cooperation with the Center for Quality Assurance and Development of the Johannes Gutenberg-University Mainz, Germany. It featured 16 items with question types including multiple choice, free field, and single choice. A total of 216 students in the third year—fifth and sixth semesters—of medical education participated in the survey. Of these, 62.0% (134/216) were female, 36.1% (78/216) were male, and 1.9% (4/216) did not disclose their gender. At the time of the survey, all students were participating in either the course General Pathology or Special Pathology. Prior to this, in their second year of medical education, all students had already completed a histological course. The voluntary survey was conducted during the first lecture of the course and included all the students of the year.

The survey aimed to investigate students’ expectations of a virtual microscope with a view to the functional needs, such as useful features (eg, points of interest, annotations) and usability questions (eg, user-friendly handling). Furthermore, their experiences with already existing WSI applications were registered. Besides establishing baseline data, including gender, age, and time-based Internet usage, items were included that covered preexisting WSI experiences by asking for prior WSI usage. In addition, students were required to specify which applications were already known. Furthermore, we asked for expectations by enumerating items, which could be chosen if considered important. Finally, a free-field item for the students’ own suggestions completed the survey. The entire set of items is given in Table 1—see Multimedia Appendix 1 for a detailed description of the variables.

**Table 1:** Descriptive analysis of the dataset of the questionnaire.

Variable^a^	Number of observations, n	Mean	SD	Kurtosis	SE
Gender	212	0.6321	0.4834	-1.7122	0.0332
Age	213	1.1455	0.3535	1.9941	0.0242
InternetAccess	207	1.2995	0.7420	4.4098	0.0516
InternetUsage	207	2.5556	0.7731	-0.4041	0.0537
InternetCompetency	205	2.3415	1.0197	1.6230	0.0712
WSIUsage	201	2.3881	1.0529	1.5121	0.0743
AdvantagesForTests	204	1.0490	0.2937	36.2616	0.0206
Workplace	200	1.4100	0.7842	0.1971	0.0555
D_PC	216	0.3194	0.4673	-1.4150	0.0318
D_Laptop	216	0.8426	0.3650	1.4978	0.0248
D_Phone	216	0.0000	0.0000	N/A^b^	0.0000
D_Tablet	216	0.0231	0.1507	37.8429	0.0103
OfflineVersion	198	1.2020	0.4025	0.1709	0.0286
UsabilityWSI	203	2.5813	1.0230	0.1626	0.0718
ImageQualityWSI	205	2.3756	1.1204	0.5234	0.0783
F_BackgroundInfo	216	0.6759	0.4691	-1.4493	0.0319
F_Forum	216	0.0417	0.2003	18.8398	0.0136
F_Lattitude	216	0.8796	0.3261	3.3850	0.0222
F_HAnnotations	216	0.8843	0.3207	3.7083	0.0218
F_PAnnotations	216	0.8611	0.3466	2.3118	0.0236
F_POI	216	0.8380	0.3693	1.3245	0.0251
F_TeachingTexts	216	0.5231	0.5006	-2.0007	0.0341
K_MHM	216	0.9213	0.2699	7.6916	0.0184
K_Histoweb	216	0.0741	0.2625	8.4730	0.0179
K_HistonetUlm	216	0.0370	0.1893	21.8072	0.0129
K_Histology	216	0.0000	0.0000	N/A	0.0000
K_HistonetMarburg	216	0.0139	0.1173	66.3673	0.0080
K_HistoWebAtlas	216	0.0185	0.1351	48.5383	0.0092
K_vMic	216	0.0139	0.1173	66.3673	0.0080
K_Pathorama	216	0.0046	0.0680	209.0277	0.0046
K_virtPatho	216	0.0046	0.0680	209.0277	0.0046
K_NeoCortex	216	0.0000	0.0000	N/A	0.0000
K_AVKurs	216	0.0000	0.0000	N/A	0.0000
K_Histologiekurs	216	0.0046	0.0680	209.0277	0.0046
K_other	216	0.0694	0.2548	9.3594	0.0173

^a^See Multimedia Appendix 1 for a detailed description of the variables.

^b^Not applicable (N/A).

### Development of the Client

The client application was implemented using JavaScript, HTML5, and Cascading Style Sheets (CSS). As JavaScript implementations differ by browser engine [18], the frameworks JQuery 1.7.1 (jQuery Foundation), MochiKit 1.4.2 (Mochi Media), and Modernizr 2.5.3 were used to abstract JavaScript code from the browser implementation. OpenLayers 2.12 was employed to display image slide data.

### Development of the Backend

To speed up application development, the backend was developed using the Web Server Gateway Interface abstraction layer. Python 2.7 (Python Software Foundation) was used as the programming language in combination with the TurboGears 2.2 framework. The database is hosted by a MySQL Server 5.1.66 (Oracle Corporation, Redwood Shores, CA).

### Slide Acquisition

We decided to use a single, virtual plane of focus throughout the physical glass slide. This method proved to be more difficult in maintaining a good focus throughout the slide than initially assumed. The autofocus functionality only provided a rough starting point resulting in the need to adjust the focal points in nearly every glass slide to achieve optimal results.

The slides were converted into image files with the slide scanner NanoZoomer 2.0-HT (C9600-13) (Hamamatsu Photonics Deutschland GmbH, Herrsching am Ammersee, Germany) producing high-quality scans. Unfortunately, the scanner software did not produce an open image format that we could use. This resulted in the necessity of converting the image files into a format we could utilize. However, in the meantime a set of tools were published to handle the Hamamatsu image format (NDPI) [19,20]. We decided to implement the Zoomify file format.

### Image File Storage

Due to the small file size of approximately 20 KB per tile, the slide image data themselves were put into the database since it would be more expensive to open a file handler for each tile than to query the database [19]. However, this yielded a backup problem. A common way to solve this problem is to dump the database at regular intervals and keep the dumps in a safe place. Because of the file size of the image data, the size of these dumps became huge. With the chosen image quality of the slides and slide dimensions, the file sizes in Pate amounted to approximately 1.5 GB to 2 GB per slide. It would have been inefficient to keep multiple backups of that size which contained mostly redundant data. As the dumps are of a text file nature, a revision control system—GIT 1.8.3.2—was installed to enable dumping of the database regularly, to store the dumps in an efficient manner by accounting only for the differences between revisions, and to permit recovery of backups from any point in time.

### Web Design

We commissioned Grüne Kommunikationsdesign, Bodenheim, Germany, for the task of Web design. The goals included design of a streamlined user experience, high usability on both mobile devices and desktops, as well as an appealing graphical interface.

## Results

### Overview

The Web application, Pate, was developed according to the expectations of medical students. The analysis of the questionnaire revealed that 97.1% (198/204) of the students considered WSI as an important learning tool in the training of histopathological skills. Furthermore, the students assessed WSI as desirable for exam preparation.

### Requested Features

**Figure 2:**
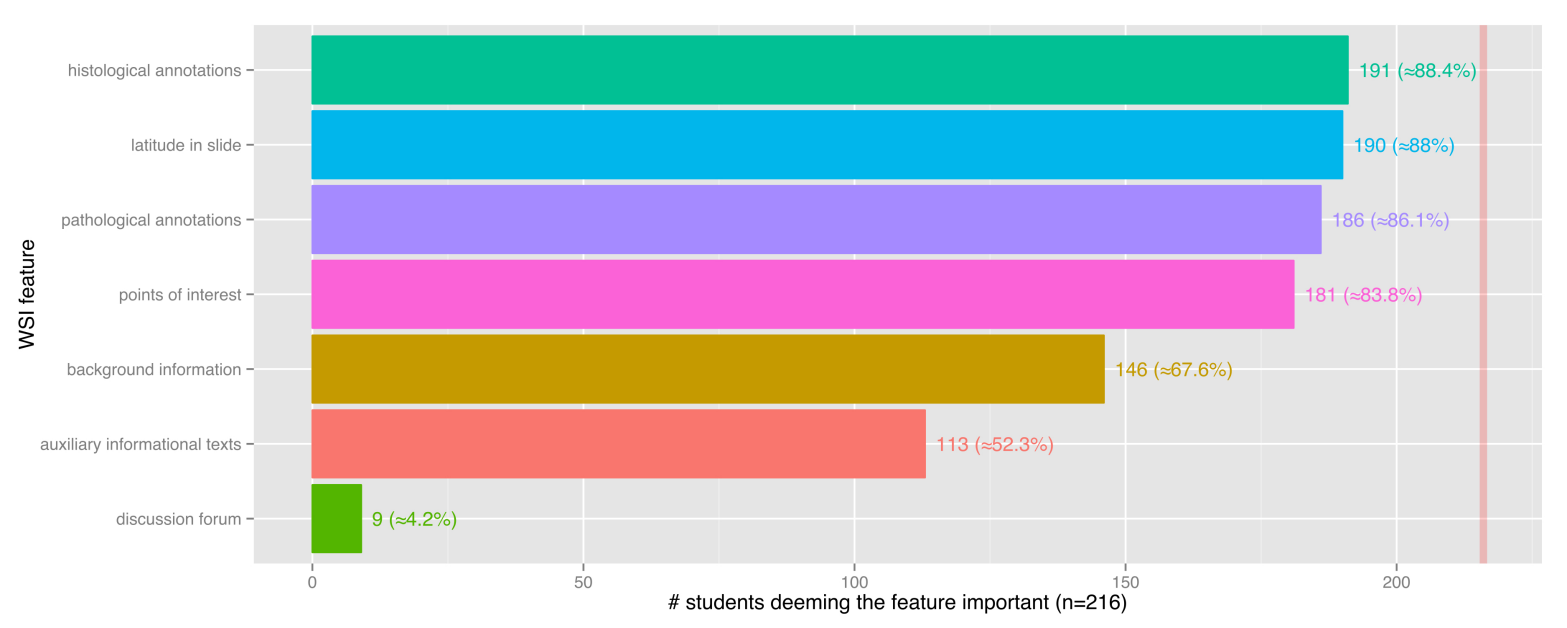
Features for WSI applications in relation to the importance to the students.

A total of 83.8% (181/216) of the students accorded a high priority to points of interest as a prime feature of Pate. Annotations—histological (191/216, 88.4%) and pathological (186/216, 86.1%)—as well as auxiliary informational texts (113/216, 52.3%) were also evaluated positively. In contrast, the deployment of a discussion forum seemed to have little importance for the students, since only 9 out of 216 students (4.2%) recommended this feature. Figure 2 shows graphical results of the importance of WSI application features by students. Furthermore, in the free-field part of the survey, a quiz mode was suggested by the students.

### Previously Known Whole-Slide Imaging Applications

**Figure 3:**
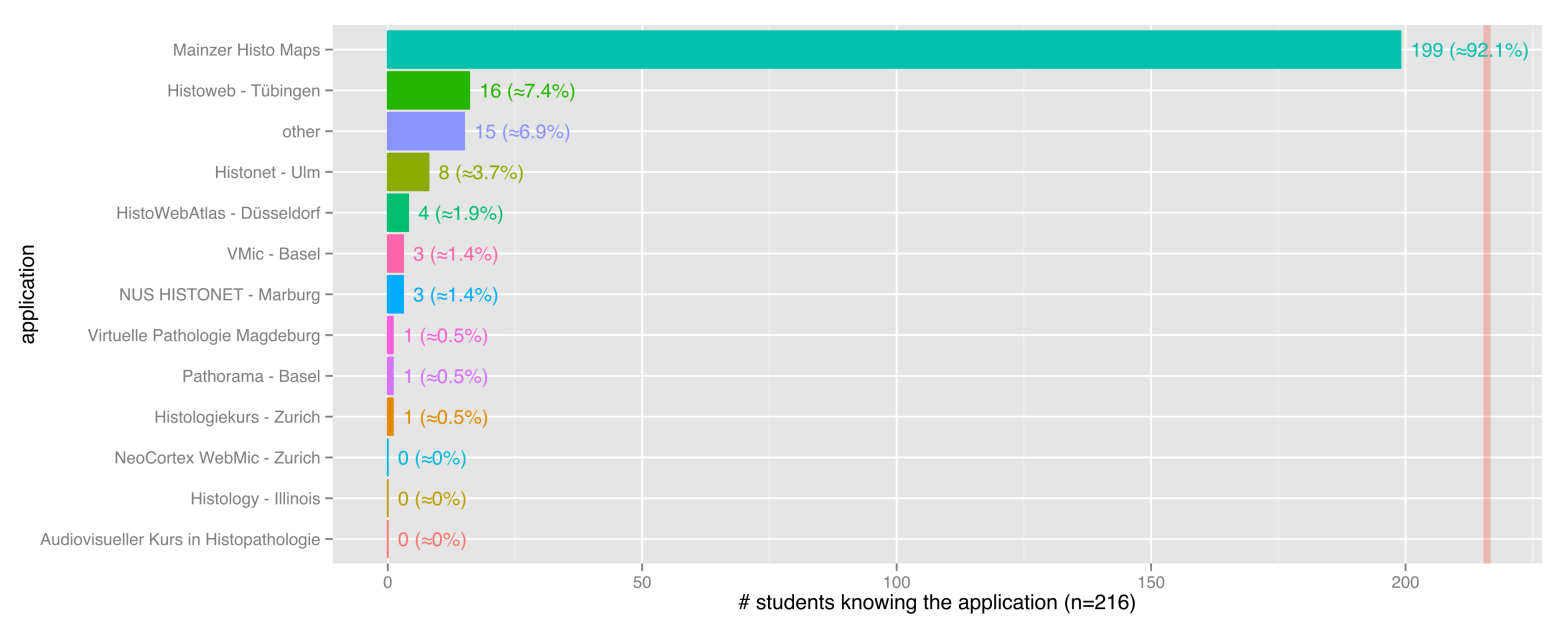
Previously known WSI applications in relation to students' degree of familiarity to these applications.

One goal of the survey was to evaluate students’ preexisting experiences with other virtual microscopes, including non-WSI systems. For this purpose, a multiple choice question was included in the survey containing an enumeration of the most known applications. As expected, since all students had already completed a histological course, which propagated this specific system, most students (199/216, 92.1%) already knew the Mainzer Histo Maps application [4], an online image collection of histological slides of different human organs. The second-most known, the Histoweb Tübingen [20], was less popular (16/216, 7.4%), followed by Histonet Ulm [21] (8/216, 3.7%). All other explicitly listed systems were known by less than 2% of the students. Of the students, 6.9% (15/216) were familiar with an application which was not listed. NYU Virtual Microscope was not part of the questionnaire since this application was not yet published at the time of our students’ survey. Figure 3 shows the graphical results of students’ familiarity with WSI applications.

### Statistical Correlation and Descriptive Analysis of the Dataset

**Figure 4:**
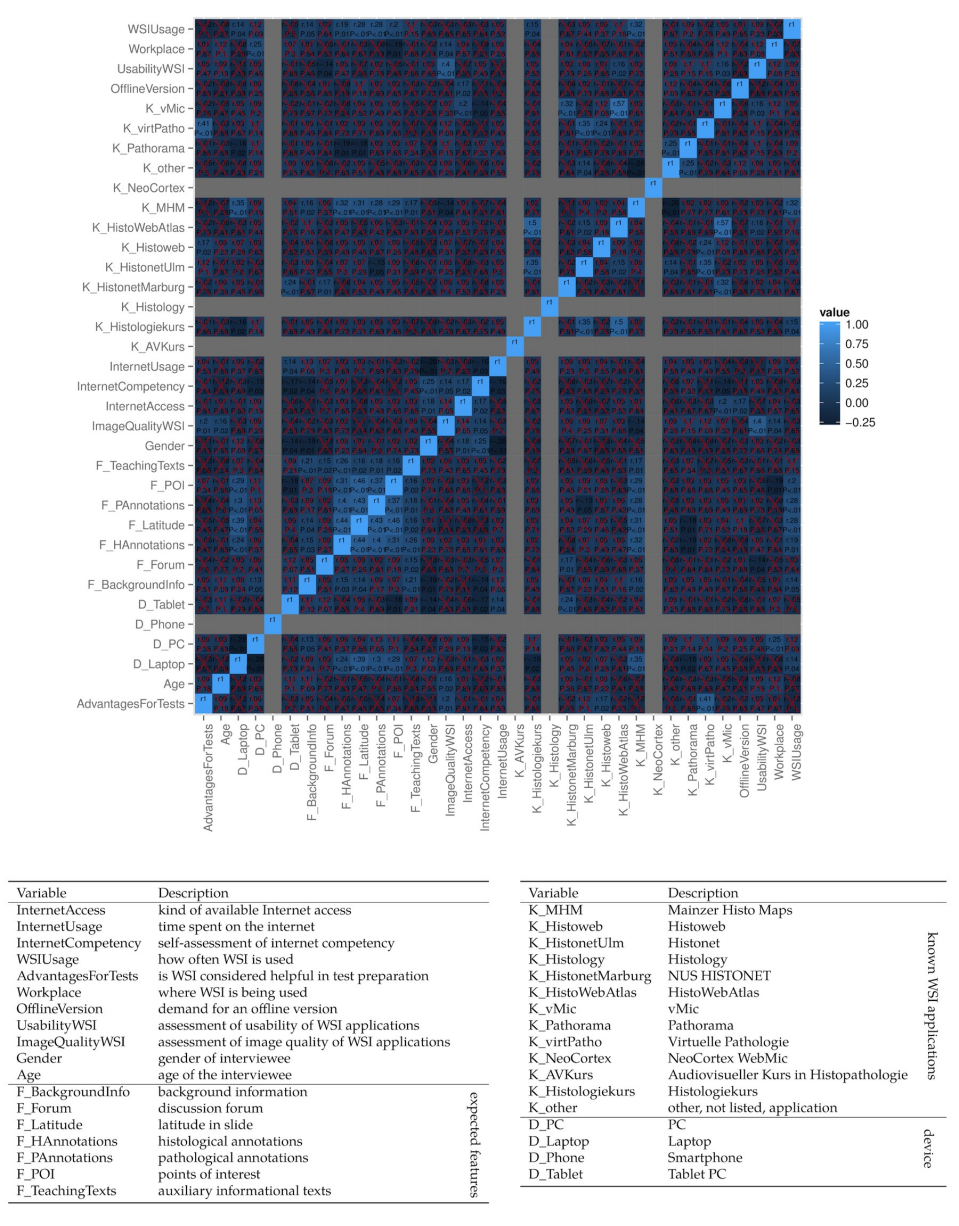
Spearman rank correlation coefficient heat map of the questionnaire items. Each rectangle contains the correlation coefficient in the upper half and the corresponding *P* value in the lower half, if applicable. A red cross marks individual *P* values where *P*<.05. The color of each rectangle indicates the value of the correlation coefficient. The legend below the heat map provides a concise description for each variable.

In order to reveal any correlations in the dataset of the questionnaire, we created a heat map for the Spearman rank correlation coefficient, ρ, or r as in Figure 4, containing every variable of the questionnaire paired with each other. The resulting figure shows all correlation coefficients and *P* values, where applicable. However, there are some variables where a correlation is not applicable, as there is no deviation in the dataset. This applies to the variables of two known WSI applications, as well as the usage of a mobile phone as a device to access Pate. Furthermore, most of the correlations are statistically insignificant (*P*>.05). However, some clusters of moderate correlation (ρ<.6) remain. For example, there appears to be an association between wanted WSI features, as well as an association between some known WSI applications.

### The New Whole-Slide Imaging Tool, Pate

The results of this survey provided the basis for the development of a novel, user-friendly application built using modern Web technologies, such as HTML5, CSS, and JavaScript. These technologies provide a unified user experience across all major platforms, such as PCs, tablets, and mobile phones. For optimal use, a modern Web browser is recommended.

**Figure 5:**
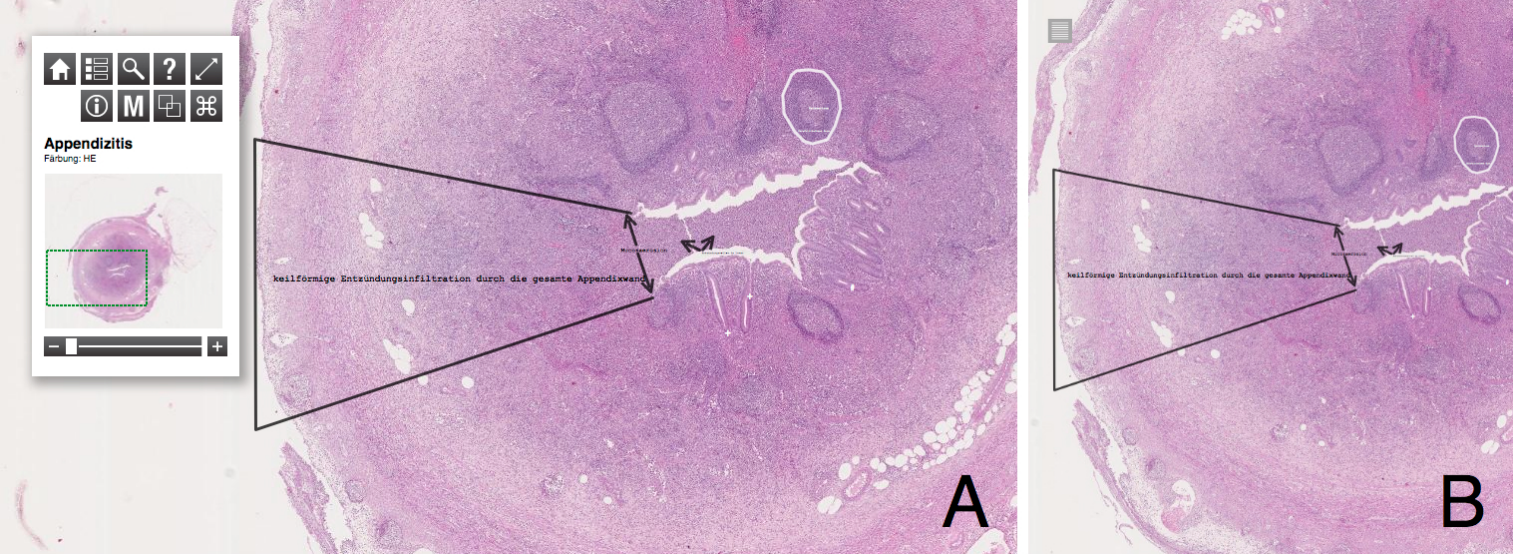
Side-by-side view of desktop layout (A) versus mobile layout (B) depicting appendicitis (ie, inflammation of the appendix). The control panel folds if the screen resolution is too small (B) to provide an unhindered view on the slide. Pathological annotations are shown.

Pate contains 118 high-quality histopathological specimens from the major human pathological conditions, enriched by several specimens regarding cell-tissue interactions. The slides showing *full-thickness cartilage defect* and *punch-biopsy skin wound* demonstrate the potential benefits of WSI applications also in biomaterial research. These slides are enriched using nondestructive annotations, as well as points of interest. Each slide in Pate can be shared by distributing the URL. This allows easy sharing of large images as soon as the slides are digitized. Utilizing modern Internet technologies, such as HTML5, CSS, and JavaScript, enables the user to view the image material with a modern Web browser—no proprietary plug-in is required. Pate supports devices with differing screen sizes, such as PCs and mobile phones, by utilizing responsive Web design methods (see Figure 5).

### Performance of Pate

**Figure 6:**
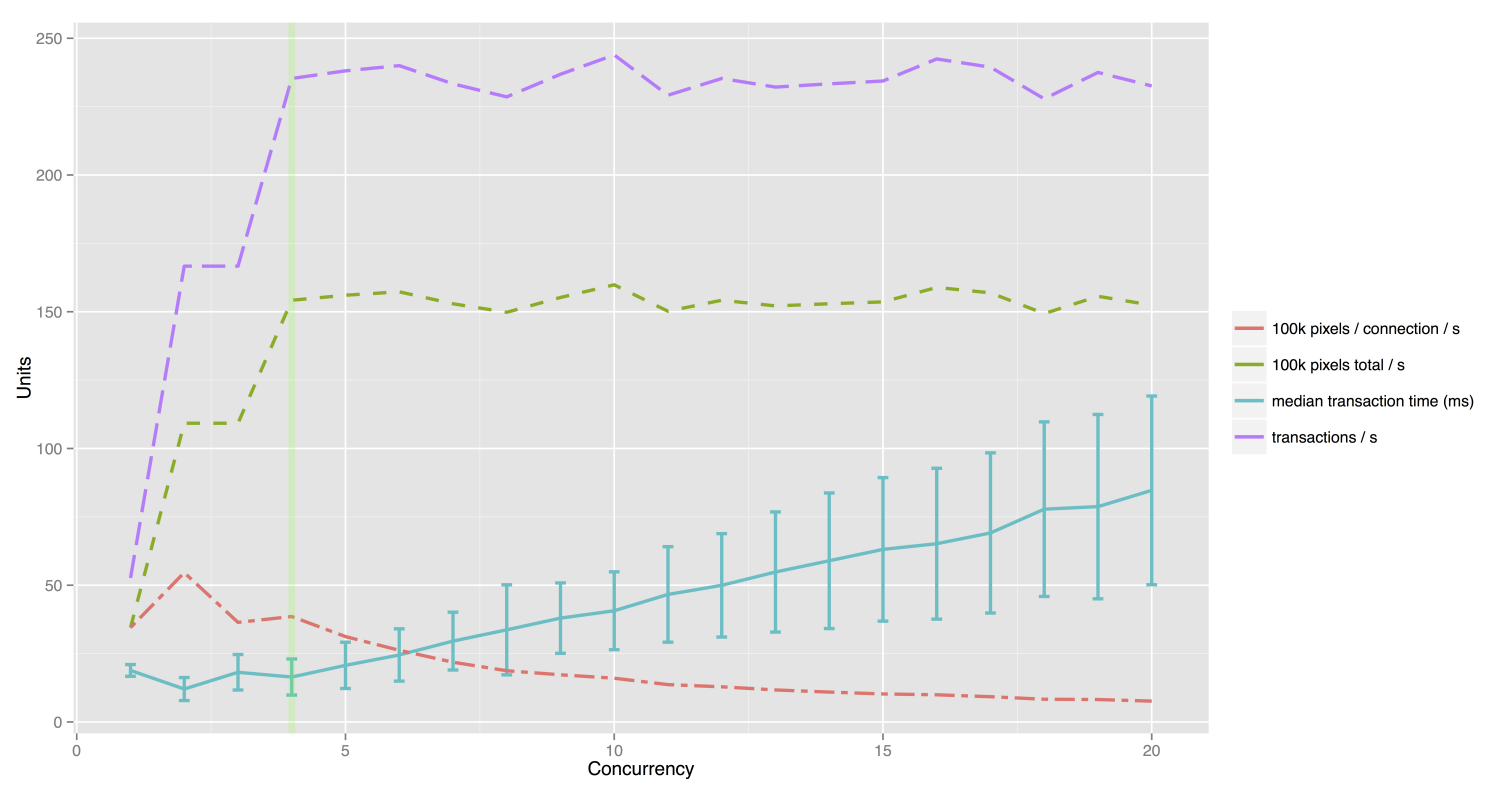
Plot of the values of Table 4 depicting the median response time in msec, including the standard deviation, as well as the transactions per second, pixels per second, and total and individual pixels per worker per second.

It is hard to determine the actual performance of the image-serving capabilities of Pate. It depends on various factors, such as the performance of the Internet service provider (ISP) of the server, the bandwidth of the ISP offered to the user, and specific routing conditions, among others [22]. Therefore, we chose a test setup which is better controlled by utilizing another computer in the same local area network as the server for testing. This allows us to reduce the influence of poor network performance. We created a list of 50 randomly sampled URLs, addressing 256x256 pixel image tiles, served by the Pate image server. All caching had been disabled. We set up Siege 2.70 (Joe Dog Software) [23], a load-testing and benchmarking tool, in order to simulate high concurrency transactions. One transaction is a complete HTTP request of one randomly sampled URL, the download of the image data, and the closing of the connection. The degree of concurrency determines how many transactions are performed in parallel. When one transaction is finished, the next one is started immediately. For each concurrency level, one worker is instantiated by Siege. Every worker performs 1000 sequentially executed transactions. For example, a concurrency level of 4 results in 4x1000 transactions, resulting in 4000 transactions. This test was performed for concurrency levels ranging from 1 to 20. The server is powered by a dual-core Intel Xeon E5504 @ 2.00 GHz CPU, 4.6 GB of RAM, and multiple hard drives in a Redundant Array of Independent Disks (RAID) 5 system with a data retrieval capacity of up to 109 MB/s. The results are shown in Table 2 and illustrated in Figure 6.

For a concurrency level of 1, the data show a median response time of 18.8 ms (SD 4.3), while performing 52.6 transactions per second. This translates to a maximum image-serving capability of 3.4 megapixels (MP) per second for a single worker. The overall performance rises up to a concurrency level of 4, resulting in a total of 235.9 transactions per second and 15.4 MP per second while achieving 3.8 MP per connection. Higher concurrency levels do not further boost the performance, but result in overall higher response time and lower individual performance per worker.

**Table 2:** Summary of the test setup of Pate.

Concurrency	Total transactions performed, n	Response time (ms), mean (SD)	Transactions/ second, mean	Pixels/ second, mean	Individual transactions/ second/ worker, mean	Individual pixels/ second/ worker, mean
1	1000	18.8 (4.3)	52.6	3,449,263	52.6	3,449,263
2	2000	12.0 (8.4)	166.7	10,922,667	83.3	5,461,333
3	3000	18.2 (13.0)	166.7	10,922,667	55.6	3,640,889
4	4000	16.4 (13.2)	235.3	15,420,235	58.8	3,855,059
5	5000	20.7 (16.9)	238.1	15,603,810	47.6	3,120,762
6	6000	24.5 (19.1)	240.0	15,728,640	40.0	2,621,440
7	7000	29.6 (21.1)	233.3	15,291,733	33.3	2,184,533
8	8000	33.7 (32.9)	228.6	14,979,657	28.6	1,872,457
9	9000	38.0 (25.7)	236.8	15,521,684	26.3	1,724,632
10	10,000	40.7 (28.5)	243.9	15,984,390	24.4	1,598,439
11	11,000	46.6 (34.9)	229.2	15,018,667	20.8	1,365,333
12	12,000	50.0 (37.8)	235.3	15,420,235	19.6	1,285,020
13	13,000	54.8 (43.9)	232.1	15,213,714	17.9	1,170,286
14	14,000	58.9 (49.6)	233.3	15,291,733	16.7	1,092,267
15	15,000	63.1 (52.5)	234.4	15,360,000	15.6	1,024,000
16	16,000	65.2 (55.2)	242.4	15,887,515	15.2	992,970
17	17,000	69.1 (58.6)	239.4	15,691,718	14.1	923,042
18	18,000	77.8 (63.9)	227.9	14,932,253	12.7	829,570
19	19,000	78.7 (67.4)	237.5	15,564,800	12.5	819,200
20	20,000	84.7 (69.0)	232.6	15,240,930	11.6	762,047

## Discussion

### Principal Findings

The goal of this project was to create a new WSI application for histopathological education according to students’ demands and expectations. Therefore, a feature set was extracted from existing WSI applications, including histological and pathological annotations, points of interest, background information, latitude in slide, teaching texts, and a discussion forum. Then, a questionnaire was designed to evaluate the actual needs of medical students, as well as their expectations and their experience with WSI applications. The results of the survey built up the base for a feature set for Pate. From a technical point of view, a further goal of the development was to utilize established Web technologies, such as HTML5 and JavaScript, in order to support as many platforms and devices as possible without the requirement to install any kind of software in advance. In addition, we put an emphasis on supporting mobile platforms with small screens and touch interfaces. This resulted in a unique set of requirements for the development of Pate that was not covered by any other application. During the development, student feedback ensured that the desired features were integrated as was intended by the results of the survey.

With the survey, we identified a high demand regarding a broad feature set for the application, including annotations, POIs, and auxiliary informational texts. We were surprised to learn that, by contrast, the students placed little emphasis on the installation of a discussion forum to permit direct contact with the staff. From the viewpoint of the organizer this facilitates the care of Pate, since a moderation of such a forum is time and personnel consuming. Nevertheless, from the viewpoint of the teacher it would be of interest to establish what problems the students are having in learning histopathological skills. Such a forum could open interesting perspectives to give live information and to react to the needs of the medical students. However, an open forum carries the risk of containing uncontrolled information, as well as incorrect data, which are not useful for an e-learning application. Therefore, the development of a discussion forum was abandoned. A statistical analysis of the survey dataset revealed no further insight besides a correlation of desired WSI features, as well as an association between some known WSI applications.

During deployment of Pate, a scripting language was used, which allowed swift response to the students’ demands and offered the implementation of a rapid application development process. By using a professional Web design, a user-friendly and intuitive Web frontend was created. In this context, we focused on supporting mobile devices, as well as conventional desktop computers.

Current limitations of Pate arise mainly as a result of the Zoomify file format used to store digital slides. This format utilizes tiles to limit the data which must be transferred to the client. This leads to approximately 200,000 files for a regular slide. The costs of retrieving a file handle for each file can be reduced by storing the image files in a database. However, storing the image data in the database complicates the backup. Commonly, time-stamped database dumps are used. This would lead to a volume of data which would be hard to handle. In Pate, this issue was solved by using a version control system. However, creating a backup would be much less difficult without the image files in the database.

One of the design goals of Pate was to support mobile devices. These devices commonly have a slow Internet connection. Because image quality was the first priority when creating the Zoomify tiles, these have a mean size of approximately 20 KB. This can lead to prolonged loading times when using a slow Internet connection.

Finally, the new Web application, Pate, is modeled closely along the expectations of the students by providing a complete set of features, such as points of interest, annotations, and informational texts, which are actually not available as a holistic feature set by other WSI applications.

### Perspectives

#### Follow-Up Survey

As Pate's development has now sufficiently progressed, we will be conducting a follow-up survey, in order to determine if the students’ needs have changed and if the implemented feature set has been implemented in a satisfactory way. Furthermore, we will establish a constant feedback loop to be able to respond to new challenges promptly.

#### Quiz Mode

The survey revealed that many students appreciated a quiz mode. Since Pate was not designed to offer tests of any kind, this feature would have to be implemented from scratch. Pate includes slide image data, meta-information, and regions of interest within each slide. Thus, views of POI regions could be generated, which the students would then be asked to diagnose.

#### Image Server

The image-serving capabilities of Pate are competitive. The median image retrieval time was measured as 18.8 ms (SD 4.3) while achieving 3.4 MP per second. The peak performance was reached with 15.4 MP per second. This puts Pate in the same performance category as other state-of-the-art image servers [24]. Most of the limitations of Pate were inherited by the Zoomify file format storing prerendered image tiles in a database. A more convenient way to handle large image data would be to archive a single file which can be stored outside the database, while allowing the user to quickly retrieve any section at any magnification. The Tagged Image File Format (TIFF) features storage of pyramidal image data.

Therefore, one future goal is to adapt or to develop an image server that is able to read pyramidal TIFF data server-side and deliver requested image tiles via the Zoomify file format to the client. This method avoids the necessity to redevelop the client and brings the benefit of a file format that supports storing slide image data in one large file. Furthermore, image data could be compressed, according to the current bandwidth, to the client, thus providing a smoother experience for users with small bandwidth, especially users on mobile networks.

### Conclusions

According to our survey, students of the University Medical Center Mainz favored a rich feature set, including WSI functionality, POIs, auxiliary informational texts, and annotations. None of the preexisting WSI applications sufficiently fitted in this profile. The feature set could be implemented by relying on rapid application development techniques and open source technologies.

Based on this survey, a new WSI application has been deployed enabling anyone with a device that features a modern Web browser to explore digital slides. This includes mobile phones like Android and iPhone, tablets, desktops, and laptops. On every platform, a suitable user interface is provided to allow the maximum potential of this tool. This could only be achieved by employing Web technologies that are supported by all modern browsers: JavaScript, HTML5, and CSS. No proprietary plug-ins like Adobe Flash are being used.

Although Pate currently has some issues with the Zoomify file format, it could be shown what Web technologies are capable of providing high-performance WSI experience.

In a future survey, we will analyze whether Pate fits within the expectations of medical students, and we will cross-compare these results with other tools known by the students.

## Abbreviations

CSS: Cascading Style Sheets
HTML5: Hypertext Markup Language 5
ISP: Internet service provider
MP: megapixel
NYUVM: NYU Virtual Microscope
POI: points of interest
RAID: Redundant Array of Independent Disks
TIFF: Tagged Image File Format
WSI: whole-slide imaging

## Multimedia Appendix 1

This ZIP file includes all information regarding the students' survey. This covers the dataset (data.csv) as well as an extensive documentation (survey_statistics.Rmd) and a compiled version (survey_statistics.html).
