# Supplementary material for: A Virtual Microscope for Academic Medical Education: The Pate Project
Source: Interact J Med Res. 2015 May 11;4(2):e11. doi: 10.2196/ijmr.3495 (PMC4443020; doi:10.2196/ijmr.3495)
Supplement: Supplementary file 1 [file ijmr_v4i2e11_app1.zip › survey_statistics/survey_statistics.html]

Statistics for the Survey of Pate


# Statistics for the Survey of Pate

We designed a survey to determine student expectations of WSI applications for teaching histological and pathological diagnosis. The resulting data set consists of 216 observations of 35 variables.

### The Survey

The survey features 16 items of the types multiple choice, free field and single choice. 216 students in the 3rd year of medical education participated in the survey. As the survey was held at a German university it was designed in the German language. We recognize, that this might not accommodate most readers. Therefore, we translated the survey:

#### Question 1

Type: single choice  
**What kind of Internet access is available at your home?**

1. broadband
2. mobile internet
3. modem
4. no internet access

Variable: InternetAccess

#### Question 2

Type: single choice  
**How much time per day do you spend in the internet?**

1. less than 30 minutes per day
2. less than 1 hour per day
3. 2 hours per day
4. 4 hours or more per day

Variable: InternetUsage

#### Question 3

Type: single choice  
**I am competent in the usage of the internet**

I fully agree <–[1]–[2]–[3]–[4]–[5]–[6]–[7]–> I strongly disagree

Variable: InternetCompetency

#### Question 4

Type: single choice  
**How often do you use virtual microscopy applications?**

1. never
2. at least once per year
3. once per month
4. once per week
5. multiple times per week
6. daily

Variable: WSIUsage

#### Question 5

Type: multiple choice  
**Which virtual microscopy applications did you use?**

| variable | application | university |
| --- | --- | --- |
| K\_MHM | Mainzer Histo Maps | Mainz |
| K\_Histoweb | Histoweb | Tübingen |
| K\_HistonetUlm | Histonet | Ulm |
| K\_Histology | Histology | Illinois |
| K\_HistonetMarburg | NUS HISTONET | Marburg |
| K\_HistoWebAtlas | HistoWebAtlas | Düsseldorf |
| K\_vMic | vMic | Basel |
| K\_Pathorama | Pathorama | Basel |
| K\_virtPatho | Virtuelle Pathologie | Magdeburg |
| K\_NeoCortex | NeoCortex WebMic | Zurich |
| K\_AVKurs | Audiovisueller Kurs in Histopathologie |  |
| K\_Histologiekurs | Histologiekurs | Zurich |
| K\_other | other, not listed, application |  |

Furthermore, additional, not listed, applications could be added in a free field. This data was scanned into image files and reviewed. However, This data is not included in the dataset, as it is not viable for analysis.

#### Question 6

Type: single choice  
**Has virtual microscopy been helpful in test preparation?**

1. yes
2. no
3. did not use virtual microscopy in test preparation

Variable: AdvantagesForTests

#### Question 7

Type: single choice  
**Where do you use virtual microscopy applications?**

1. at home
2. at the university
3. at home and at the university

Variable: Workplace

#### Question 8

Type: multiple choice  
**What devices do you use for virtual microscopy?**

| variable | device |
| --- | --- |
| D\_PC | PC |
| D\_Laptop | Laptop |
| D\_Phone | Smartphone |
| D\_Tablet | Tablet PC |

#### Question 9

Type: single choice  
**Do you wish for an offline version of the application?**

1. yes
2. no

Variable: OfflineVersion

#### Question 10

Type: single choice  
**What do you think about the usability of existing virtual microscopy applications?**

very easy <–[1]–[2]–[3]–[4]–[5]–[6]–[7]–> very hard

Variable: UsabilityWSI

#### Question 11

Type: single choice  
**What do you think about the image quality of existing virtual microscopy applications?**

very good <–[1]–[2]–[3]–[4]–[5]–[6]–[7]–> very bad

Variable: ImageQualityWSI

#### Question 12

Type: multiple choice  
**What features would you expect from a virtual microscopy application?**

| variable | feature |
| --- | --- |
| F\_BackgroundInfo | background information |
| F\_Forum | discussion forum |
| F\_Latitude | latitude in slide |
| F\_HAnnotations | histological annotations |
| F\_PAnnotations | pathological annotations |
| F\_POI | points of interest |
| F\_TeachingTexts | auxiliary informational texts |

Furthermore, additional, not listed, features could be added in a free field. This data was scanned into image files and reviewed. However, This data is not included in the data set, as it is not viable for analysis.

#### Question 13

Type: free field  
**What are the TOPS of virtual microscopy applications that you have used?**

This data was scanned into image files and reviewed. However, This data is not included in the data set, as it is not viable for analysis.

Variable: None

#### Question 14

Type: free field  
**What are the FLOPS of virtual microscopy applications that you have used?**

This data was scanned into image files and reviewed. However, This data is not included in the data set, as it is not viable for analysis.

Variable: None

#### Question 15

Type: single choice  
**What is your gender?**

1. male
2. female

Variable: Gender

#### Question 16

Type: single choice  
**What is your age?**

1. 20-25 years
2. 26-30 years
3. 31 years or older

Variable: Age

### Descriptive Statistics of the Dataset

|  | vars | n | mean | sd | skew | kurtosis | se |
| --- | --- | --- | --- | --- | --- | --- | --- |
| Gender | 1 | 212 | 0.6321 | 0.4834 | -0.5439 | -1.7122 | 0.0332 |
| Age | 2 | 213 | 1.1455 | 0.3535 | 1.9962 | 1.9941 | 0.0242 |
| InternetAccess | 3 | 207 | 1.2995 | 0.7420 | 2.3708 | 4.4098 | 0.0516 |
| InternetUsage | 4 | 207 | 2.5556 | 0.7731 | -0.0594 | -0.4041 | 0.0537 |
| InternetCompetency | 5 | 205 | 2.3415 | 1.0197 | 1.0793 | 1.6230 | 0.0712 |
| WSIUsage | 6 | 201 | 2.3881 | 1.0529 | 1.4837 | 1.5121 | 0.0743 |
| AdvantagesForTests | 7 | 204 | 1.0490 | 0.2937 | 6.0762 | 36.2616 | 0.0206 |
| Workplace | 8 | 200 | 1.4100 | 0.7842 | 1.4483 | 0.1971 | 0.0555 |
| D\_PC | 9 | 216 | 0.3194 | 0.4673 | 0.7691 | -1.4150 | 0.0318 |
| D\_Laptop | 10 | 216 | 0.8426 | 0.3650 | -1.8684 | 1.4978 | 0.0248 |
| D\_Phone | 11 | 216 | 0.0000 | 0.0000 | NaN | NaN | 0.0000 |
| D\_Tablet | 12 | 216 | 0.0231 | 0.1507 | 6.2982 | 37.8429 | 0.0103 |
| OfflineVersion | 13 | 198 | 1.2020 | 0.4025 | 1.4731 | 0.1709 | 0.0286 |
| UsabilityWSI | 14 | 203 | 2.5813 | 1.0230 | 0.5957 | 0.1626 | 0.0718 |
| ImageQualityWSI | 15 | 205 | 2.3756 | 1.1204 | 0.8726 | 0.5234 | 0.0783 |
| F\_BackgroundInfo | 16 | 216 | 0.6759 | 0.4691 | -0.7466 | -1.4493 | 0.0319 |
| F\_Forum | 17 | 216 | 0.0417 | 0.2003 | 4.5555 | 18.8398 | 0.0136 |
| F\_Latitude | 18 | 216 | 0.8796 | 0.3261 | -2.3172 | 3.3850 | 0.0222 |
| F\_HAnnotations | 19 | 216 | 0.8843 | 0.3207 | -2.3856 | 3.7083 | 0.0218 |
| F\_PAnnotations | 20 | 216 | 0.8611 | 0.3466 | -2.0739 | 2.3118 | 0.0236 |
| F\_POI | 21 | 216 | 0.8380 | 0.3693 | -1.8216 | 1.3245 | 0.0251 |
| F\_TeachingTexts | 22 | 216 | 0.5231 | 0.5006 | -0.0920 | -2.0007 | 0.0341 |
| K\_MHM | 23 | 216 | 0.9213 | 0.2699 | -3.1074 | 7.6916 | 0.0184 |
| K\_Histoweb | 24 | 216 | 0.0741 | 0.2625 | 3.2301 | 8.4730 | 0.0179 |
| K\_HistonetUlm | 25 | 216 | 0.0370 | 0.1893 | 4.8689 | 21.8072 | 0.0129 |
| K\_Histology | 26 | 216 | 0.0000 | 0.0000 | NaN | NaN | 0.0000 |
| K\_HistonetMarburg | 27 | 216 | 0.0139 | 0.1173 | 8.2498 | 66.3673 | 0.0080 |
| K\_HistoWebAtlas | 28 | 216 | 0.0185 | 0.1351 | 7.0932 | 48.5383 | 0.0092 |
| K\_vMic | 29 | 216 | 0.0139 | 0.1173 | 8.2498 | 66.3673 | 0.0080 |
| K\_Pathorama | 30 | 216 | 0.0046 | 0.0680 | 14.4934 | 209.0277 | 0.0046 |
| K\_virtPatho | 31 | 216 | 0.0046 | 0.0680 | 14.4934 | 209.0277 | 0.0046 |
| K\_NeoCortex | 32 | 216 | 0.0000 | 0.0000 | NaN | NaN | 0.0000 |
| K\_AVKurs | 33 | 216 | 0.0000 | 0.0000 | NaN | NaN | 0.0000 |
| K\_Histologiekurs | 34 | 216 | 0.0046 | 0.0680 | 14.4934 | 209.0277 | 0.0046 |
| K\_other | 35 | 216 | 0.0694 | 0.2548 | 3.3639 | 9.3594 | 0.0173 |

### Heatmap of a Spearman Correlation of the Dataset

|  | Gender | Age | InternetAccess | InternetUsage | InternetCompetency | WSIUsage | AdvantagesForTests | Workplace | D\_PC | D\_Laptop | D\_Phone | D\_Tablet | OfflineVersion | UsabilityWSI | ImageQualityWSI | F\_BackgroundInfo | F\_Forum | F\_Latitude | F\_HAnnotations | F\_PAnnotations | F\_POI | F\_TeachingTexts | K\_MHM | K\_Histoweb | K\_HistonetUlm | K\_Histology | K\_HistonetMarburg | K\_HistoWebAtlas | K\_vMic | K\_Pathorama | K\_virtPatho | K\_NeoCortex | K\_AVKurs | K\_Histologiekurs | K\_other |
| --- | --- | --- | --- | --- | --- | --- | --- | --- | --- | --- | --- | --- | --- | --- | --- | --- | --- | --- | --- | --- | --- | --- | --- | --- | --- | --- | --- | --- | --- | --- | --- | --- | --- | --- | --- |
| Gender | 1.0000 | 0.0040 | 0.1492 | -0.2579 | 0.2543 | -0.0467 | -0.1144 | -0.0285 | -0.0754 | 0.1155 | NA | -0.1393 | -0.0260 | 0.0371 | -0.0348 | -0.1890 | -0.1304 | 0.0052 | 0.0850 | -0.0087 | 0.0232 | 0.0237 | -0.0452 | -0.1328 | -0.0029 | NA | -0.0742 | -0.0380 | -0.0742 | -0.0902 | -0.0902 | NA | NA | -0.0902 | -0.0334 |
| Age | 0.0040 | 1.0000 | 0.0041 | -0.0314 | -0.1083 | -0.0690 | 0.0750 | 0.0831 | 0.0272 | -0.1176 | NA | 0.1119 | -0.0907 | 0.1113 | 0.1920 | 0.1150 | -0.0205 | -0.0495 | -0.0150 | -0.0364 | 0.0034 | -0.0802 | -0.0750 | 0.0844 | -0.0115 | NA | 0.0636 | -0.0571 | -0.0493 | -0.0283 | -0.0283 | NA | NA | -0.0283 | -0.0557 |
| InternetAccess | 0.1492 | 0.0041 | 1.0000 | -0.0888 | 0.1811 | -0.0627 | -0.0465 | -0.0073 | 0.0827 | -0.0436 | NA | -0.0568 | 0.1600 | -0.0668 | 0.1257 | -0.1015 | -0.0223 | -0.0904 | 0.0392 | 0.0404 | -0.0199 | 0.0516 | -0.0295 | -0.0627 | -0.0811 | NA | 0.0055 | 0.0055 | 0.0935 | -0.0282 | -0.0282 | NA | NA | -0.0282 | -0.0310 |
| InternetUsage | -0.2579 | -0.0314 | -0.0888 | 1.0000 | -0.1648 | 0.0901 | 0.0681 | 0.1030 | -0.0224 | 0.0652 | NA | 0.1315 | -0.0888 | -0.0921 | -0.0276 | 0.1235 | 0.0831 | 0.0363 | 0.0261 | -0.0186 | -0.0357 | -0.0335 | -0.0261 | 0.0495 | 0.0506 | NA | 0.0699 | -0.0101 | -0.0349 | 0.0402 | 0.0402 | NA | NA | 0.0402 | 0.0305 |
| InternetCompetency | 0.2543 | -0.1083 | 0.1811 | -0.1648 | 1.0000 | -0.0236 | -0.0391 | -0.0934 | -0.1360 | -0.0374 | NA | -0.1463 | -0.1208 | 0.0445 | 0.0830 | -0.1279 | -0.0485 | -0.1390 | -0.0269 | -0.0201 | -0.1139 | 0.0173 | -0.0714 | -0.0798 | -0.0429 | NA | -0.0808 | -0.0127 | -0.1208 | 0.0453 | -0.0923 | NA | NA | -0.0235 | -0.0759 |
| WSIUsage | -0.0467 | -0.0690 | -0.0627 | 0.0901 | -0.0236 | 1.0000 | -0.0994 | -0.0762 | 0.1122 | 0.0883 | NA | -0.0590 | -0.0155 | 0.0686 | -0.0504 | 0.1305 | 0.0345 | 0.2221 | 0.1295 | 0.2444 | 0.1541 | 0.0462 | 0.2312 | 0.0573 | 0.0944 | NA | -0.0370 | 0.1169 | 0.0107 | 0.0412 | -0.0261 | NA | NA | 0.2432 | 0.0032 |
| AdvantagesForTests | -0.1144 | 0.0750 | -0.0465 | 0.0681 | -0.0391 | -0.0994 | 1.0000 | -0.0135 | 0.0592 | -0.1576 | NA | -0.0265 | -0.0419 | 0.1185 | 0.2866 | 0.0329 | -0.0359 | -0.0706 | -0.0101 | -0.1136 | 0.0639 | -0.1156 | -0.1386 | 0.2001 | 0.1386 | NA | -0.0204 | -0.0237 | -0.0204 | -0.0117 | 0.4674 | NA | NA | -0.0117 | -0.0454 |
| Workplace | -0.0285 | 0.0831 | -0.0073 | 0.1030 | -0.0934 | -0.0762 | -0.0135 | 1.0000 | 0.2303 | -0.0315 | NA | 0.0798 | -0.0233 | 0.1265 | 0.0964 | 0.0027 | -0.0418 | -0.0007 | -0.0236 | -0.0810 | -0.1670 | -0.0164 | -0.0235 | -0.0603 | -0.0418 | NA | 0.0405 | -0.0292 | 0.0931 | -0.0372 | -0.0372 | NA | NA | -0.0372 | 0.0566 |
| D\_PC | -0.0754 | 0.0272 | 0.0827 | -0.0224 | -0.1360 | 0.1122 | 0.0592 | 0.2303 | 1.0000 | -0.2764 | NA | -0.0394 | 0.0826 | 0.0662 | 0.0562 | 0.1350 | 0.0559 | 0.0398 | 0.0616 | 0.1316 | 0.1127 | -0.0417 | 0.0896 | 0.0337 | -0.0292 | NA | 0.0035 | 0.0532 | 0.0884 | 0.0995 | 0.0995 | NA | NA | 0.0995 | 0.0863 |
| D\_Laptop | 0.1155 | -0.1176 | -0.0436 | 0.0652 | -0.0374 | 0.0883 | -0.1576 | -0.0315 | -0.2764 | 1.0000 | NA | -0.0180 | -0.0623 | -0.1010 | -0.0270 | 0.0810 | 0.0265 | 0.3871 | 0.2410 | 0.3043 | 0.2929 | 0.0709 | 0.3458 | 0.0737 | 0.0175 | NA | 0.0513 | -0.0349 | 0.0513 | -0.1578 | 0.0295 | NA | NA | -0.1578 | -0.0820 |
| D\_Phone | NA | NA | NA | NA | NA | NA | NA | NA | NA | NA | NA | NA | NA | NA | NA | NA | NA | NA | NA | NA | NA | NA | NA | NA | NA | NA | NA | NA | NA | NA | NA | NA | NA | NA | NA |
| D\_Tablet | -0.1393 | 0.1119 | -0.0568 | 0.1315 | -0.1463 | -0.0590 | -0.0265 | 0.0798 | -0.0394 | -0.0180 | NA | 1.0000 | -0.0810 | -0.0282 | -0.0531 | 0.1066 | 0.1220 | 0.0569 | -0.0405 | -0.0272 | -0.1830 | 0.0853 | 0.0450 | -0.0435 | -0.0302 | NA | 0.2448 | -0.0211 | -0.0183 | -0.0105 | -0.0105 | NA | NA | -0.0105 | 0.0791 |
| OfflineVersion | -0.0260 | -0.0907 | 0.1600 | -0.0888 | -0.1208 | -0.0155 | -0.0419 | -0.0233 | 0.0826 | -0.0623 | NA | -0.0810 | 1.0000 | -0.1106 | -0.0270 | -0.0413 | 0.0110 | 0.0965 | -0.0936 | 0.0099 | 0.0327 | -0.0309 | -0.0245 | -0.0107 | 0.0245 | NA | -0.0624 | -0.0722 | -0.0624 | -0.0358 | -0.0358 | NA | NA | -0.0358 | 0.1206 |
| UsabilityWSI | 0.0371 | 0.1113 | -0.0668 | -0.0921 | 0.0445 | 0.0686 | 0.1185 | 0.1265 | 0.0662 | -0.1010 | NA | -0.0282 | -0.1106 | 1.0000 | 0.4437 | -0.0330 | -0.1461 | -0.0718 | 0.0502 | 0.0425 | -0.0470 | 0.0062 | 0.0409 | 0.0125 | 0.0831 | NA | 0.0102 | 0.1624 | 0.1703 | 0.0978 | 0.0978 | NA | NA | 0.0289 | 0.0545 |
| ImageQualityWSI | -0.0348 | 0.1920 | 0.1257 | -0.0276 | 0.0830 | -0.0504 | 0.2866 | 0.0964 | 0.0562 | -0.0270 | NA | -0.0531 | -0.0270 | 0.4437 | 1.0000 | -0.0163 | -0.0507 | -0.1205 | 0.0164 | -0.0592 | -0.0111 | 0.0290 | -0.1577 | 0.1300 | 0.0675 | NA | 0.0681 | 0.0473 | 0.1044 | 0.1018 | 0.1644 | NA | NA | -0.0235 | 0.0474 |
| F\_BackgroundInfo | -0.1890 | 0.1150 | -0.1015 | 0.1235 | -0.1279 | 0.1305 | 0.0329 | 0.0027 | 0.1350 | 0.0810 | NA | 0.1066 | -0.0413 | -0.0330 | -0.0163 | 1.0000 | 0.0454 | 0.1390 | 0.1515 | 0.0938 | 0.0713 | 0.2103 | 0.1650 | 0.0448 | 0.0834 | NA | -0.0023 | 0.0951 | -0.0023 | 0.0472 | 0.0472 | NA | NA | 0.0472 | 0.0335 |
| F\_Forum | -0.1304 | -0.0205 | -0.0223 | 0.0831 | -0.0485 | 0.0345 | -0.0359 | -0.0418 | 0.0559 | 0.0265 | NA | 0.1220 | 0.0110 | -0.1461 | -0.0507 | 0.0454 | 1.0000 | 0.0771 | 0.0754 | 0.0167 | 0.0917 | 0.1527 | 0.0609 | -0.0590 | -0.0409 | NA | 0.1732 | -0.0286 | -0.0247 | -0.0142 | -0.0142 | NA | NA | -0.0142 | -0.0570 |
| F\_Latitude | 0.0052 | -0.0495 | -0.0904 | 0.0363 | -0.1390 | 0.2221 | -0.0706 | -0.0007 | 0.0398 | 0.3871 | NA | 0.0569 | 0.0965 | -0.0718 | -0.1205 | 0.1390 | 0.0771 | 1.0000 | 0.4443 | 0.4274 | 0.4551 | 0.1596 | 0.3146 | 0.0503 | 0.0725 | NA | 0.0439 | -0.0547 | 0.0439 | -0.1844 | 0.0252 | NA | NA | 0.0252 | 0.0451 |
| F\_HAnnotations | 0.0850 | -0.0150 | 0.0392 | 0.0261 | -0.0269 | 0.1295 | -0.0101 | -0.0236 | 0.0616 | 0.2410 | NA | -0.0405 | -0.0936 | 0.0502 | 0.0164 | 0.1515 | 0.0754 | 0.4443 | 1.0000 | 0.3987 | 0.3122 | 0.2630 | 0.3242 | 0.0471 | 0.0710 | NA | -0.0807 | 0.0497 | -0.0807 | -0.1885 | 0.0247 | NA | NA | 0.0247 | -0.0150 |
| F\_PAnnotations | -0.0087 | -0.0364 | 0.0404 | -0.0186 | -0.0201 | 0.2444 | -0.1136 | -0.0810 | 0.1316 | 0.3043 | NA | -0.0272 | 0.0099 | 0.0425 | -0.0592 | 0.0938 | 0.0167 | 0.4274 | 0.3987 | 1.0000 | 0.3683 | 0.1794 | 0.2803 | 0.0114 | -0.1339 | NA | 0.0477 | 0.0552 | 0.0477 | 0.0274 | 0.0274 | NA | NA | 0.0274 | -0.0483 |
| F\_POI | 0.0232 | 0.0034 | -0.0199 | -0.0357 | -0.1139 | 0.1541 | 0.0639 | -0.1670 | 0.1127 | 0.2929 | NA | -0.1830 | 0.0327 | -0.0470 | -0.0111 | 0.0713 | 0.0917 | 0.4551 | 0.3122 | 0.3683 | 1.0000 | 0.1587 | 0.2914 | 0.0764 | 0.0862 | NA | 0.0522 | -0.0328 | 0.0522 | 0.0300 | 0.0300 | NA | NA | 0.0300 | -0.0281 |
| F\_TeachingTexts | 0.0237 | -0.0802 | 0.0516 | -0.0335 | 0.0173 | 0.0462 | -0.1156 | -0.0164 | -0.0417 | 0.0709 | NA | 0.0853 | -0.0309 | 0.0062 | 0.0290 | 0.2103 | 0.1527 | 0.1596 | 0.2630 | 0.1794 | 0.1587 | 1.0000 | 0.1684 | -0.0485 | -0.0582 | NA | -0.0451 | -0.0064 | -0.0451 | 0.0651 | -0.0714 | NA | NA | -0.0714 | -0.0309 |
| K\_MHM | -0.0452 | -0.0750 | -0.0295 | -0.0261 | -0.0714 | 0.2312 | -0.1386 | -0.0235 | 0.0896 | 0.3458 | NA | 0.0450 | -0.0245 | 0.0409 | -0.1577 | 0.1650 | 0.0609 | 0.3146 | 0.3242 | 0.2803 | 0.2914 | 0.1684 | 1.0000 | 0.0170 | 0.0573 | NA | -0.1122 | 0.0401 | 0.0347 | 0.0199 | 0.0199 | NA | NA | 0.0199 | -0.2583 |
| K\_Histoweb | -0.1328 | 0.0844 | -0.0627 | 0.0495 | -0.0798 | 0.0573 | 0.2001 | -0.0603 | 0.0337 | 0.0737 | NA | -0.0435 | -0.0107 | 0.0125 | 0.1300 | 0.0448 | -0.0590 | 0.0503 | 0.0471 | 0.0114 | 0.0764 | -0.0485 | 0.0170 | 1.0000 | 0.0381 | NA | -0.0336 | 0.0923 | 0.1175 | -0.0193 | 0.2411 | NA | NA | -0.0193 | -0.0773 |
| K\_HistonetUlm | -0.0029 | -0.0115 | -0.0811 | 0.0506 | -0.0429 | 0.0944 | 0.1386 | -0.0418 | -0.0292 | 0.0175 | NA | -0.0302 | 0.0245 | 0.0831 | 0.0675 | 0.0834 | -0.0409 | 0.0725 | 0.0710 | -0.1339 | 0.0862 | -0.0582 | 0.0573 | 0.0381 | 1.0000 | NA | -0.0233 | 0.1549 | -0.0233 | -0.0134 | 0.3478 | NA | NA | 0.3478 | 0.1393 |
| K\_Histology | NA | NA | NA | NA | NA | NA | NA | NA | NA | NA | NA | NA | NA | NA | NA | NA | NA | NA | NA | NA | NA | NA | NA | NA | NA | NA | NA | NA | NA | NA | NA | NA | NA | NA | NA |
| K\_HistonetMarburg | -0.0742 | 0.0636 | 0.0055 | 0.0699 | -0.0808 | -0.0370 | -0.0204 | 0.0405 | 0.0035 | 0.0513 | NA | 0.2448 | -0.0624 | 0.0102 | 0.0681 | -0.0023 | 0.1732 | 0.0439 | -0.0807 | 0.0477 | 0.0522 | -0.0451 | -0.1122 | -0.0336 | -0.0233 | NA | 1.0000 | -0.0163 | 0.3239 | -0.0081 | -0.0081 | NA | NA | -0.0081 | -0.0324 |
| K\_HistoWebAtlas | -0.0380 | -0.0571 | 0.0055 | -0.0101 | -0.0127 | 0.1169 | -0.0237 | -0.0292 | 0.0532 | -0.0349 | NA | -0.0211 | -0.0722 | 0.1624 | 0.0473 | 0.0951 | -0.0286 | -0.0547 | 0.0497 | 0.0552 | -0.0328 | -0.0064 | 0.0401 | 0.0923 | 0.1549 | NA | -0.0163 | 1.0000 | 0.5706 | -0.0094 | -0.0094 | NA | NA | 0.4965 | -0.0375 |
| K\_vMic | -0.0742 | -0.0493 | 0.0935 | -0.0349 | -0.1208 | 0.0107 | -0.0204 | 0.0931 | 0.0884 | 0.0513 | NA | -0.0183 | -0.0624 | 0.1703 | 0.1044 | -0.0023 | -0.0247 | 0.0439 | -0.0807 | 0.0477 | 0.0522 | -0.0451 | 0.0347 | 0.1175 | -0.0233 | NA | 0.3239 | 0.5706 | 1.0000 | -0.0081 | -0.0081 | NA | NA | -0.0081 | -0.0324 |
| K\_Pathorama | -0.0902 | -0.0283 | -0.0282 | 0.0402 | 0.0453 | 0.0412 | -0.0117 | -0.0372 | 0.0995 | -0.1578 | NA | -0.0105 | -0.0358 | 0.0978 | 0.1018 | 0.0472 | -0.0142 | -0.1844 | -0.1885 | 0.0274 | 0.0300 | 0.0651 | 0.0199 | -0.0193 | -0.0134 | NA | -0.0081 | -0.0094 | -0.0081 | 1.0000 | -0.0047 | NA | NA | -0.0047 | 0.2497 |
| K\_virtPatho | -0.0902 | -0.0283 | -0.0282 | 0.0402 | -0.0923 | -0.0261 | 0.4674 | -0.0372 | 0.0995 | 0.0295 | NA | -0.0105 | -0.0358 | 0.0978 | 0.1644 | 0.0472 | -0.0142 | 0.0252 | 0.0247 | 0.0274 | 0.0300 | -0.0714 | 0.0199 | 0.2411 | 0.3478 | NA | -0.0081 | -0.0094 | -0.0081 | -0.0047 | 1.0000 | NA | NA | -0.0047 | -0.0186 |
| K\_NeoCortex | NA | NA | NA | NA | NA | NA | NA | NA | NA | NA | NA | NA | NA | NA | NA | NA | NA | NA | NA | NA | NA | NA | NA | NA | NA | NA | NA | NA | NA | NA | NA | NA | NA | NA | NA |
| K\_AVKurs | NA | NA | NA | NA | NA | NA | NA | NA | NA | NA | NA | NA | NA | NA | NA | NA | NA | NA | NA | NA | NA | NA | NA | NA | NA | NA | NA | NA | NA | NA | NA | NA | NA | NA | NA |
| K\_Histologiekurs | -0.0902 | -0.0283 | -0.0282 | 0.0402 | -0.0235 | 0.2432 | -0.0117 | -0.0372 | 0.0995 | -0.1578 | NA | -0.0105 | -0.0358 | 0.0289 | -0.0235 | 0.0472 | -0.0142 | 0.0252 | 0.0247 | 0.0274 | 0.0300 | -0.0714 | 0.0199 | -0.0193 | 0.3478 | NA | -0.0081 | 0.4965 | -0.0081 | -0.0047 | -0.0047 | NA | NA | 1.0000 | -0.0186 |
| K\_other | -0.0334 | -0.0557 | -0.0310 | 0.0305 | -0.0759 | 0.0032 | -0.0454 | 0.0566 | 0.0863 | -0.0820 | NA | 0.0791 | 0.1206 | 0.0545 | 0.0474 | 0.0335 | -0.0570 | 0.0451 | -0.0150 | -0.0483 | -0.0281 | -0.0309 | -0.2583 | -0.0773 | 0.1393 | NA | -0.0324 | -0.0375 | -0.0324 | 0.2497 | -0.0186 | NA | NA | -0.0186 | 1.0000 |

```
library(Hmisc)

abbreviateSTR <- function(value, prefix){  # format string for more concisely
  lst = c()
  for (item in value) {
    if (is.nan(item) || is.na(item)) {
      lst <- c(lst, '')
      next
    }
    item <- round(item, 2)
    if (item == 0) {
      item = '<.01'
    }
    item <- as.character(item)
    item <- sub("(^[0])+", "", item)    # remove leading 0: 0.05 -> .05
    item <- sub("(^-[0])+", "-", item)  # remove leading -0: -0.05 -> -.05
    lst <- c(lst, paste(prefix, item, sep = ""))
  }
  return(lst)
}

cormatrix = rcorr(as.matrix(d), type='spearman')
cordata = melt(cormatrix$r)
cordata$labelr = abbreviateSTR(melt(cormatrix$r)$value, 'r')
cordata$labelP = abbreviateSTR(melt(cormatrix$P)$value, 'P')
cordata$label = paste(cordata$labelr, "\n", 
                      cordata$labelP, sep = "")
cordata$strike = ""
cordata$strike[cormatrix$P > 0.05] = "X"

ggplot(cordata, aes(x=Var1, y=Var2, fill=value)) + geom_tile() + 
  theme(axis.text.x = element_text(angle=90, hjust=TRUE)) +
  xlab("") + ylab("") + 
  geom_text(label=cordata$label, size=2) + 
  geom_text(label=cordata$strike, size=7, color="red", alpha=0.4)
```

### Requested Features

```
features = data.frame(feature=character(), n=numeric(), stringsAsFactors=FALSE)
features[nrow(features)+1,] <- list('histological annotations', sum(d$F_HAnnotations))
features[nrow(features)+1,] <- list('latitude in slide', sum(d$F_Latitude))
features[nrow(features)+1,] <- list('pathological annotations', sum(d$F_PAnnotations))
features[nrow(features)+1,] <- list('points of interest', sum(d$F_POI))
features[nrow(features)+1,] <- list('background information', sum(d$F_BackgroundInfo))
features[nrow(features)+1,] <- 
  list('auxiliary informational texts', sum(d$F_TeachingTexts))
features[nrow(features)+1,] <- list('discussion forum', sum(d$F_Forum))
features$label = paste(features$n, 
                       " (≈", round(features$n/nrow(d)*100, 1), "%)", sep = "")

ggplot(features, aes(x=reorder(feature, n), y=n, color=feature, fill=feature)) + 
  geom_bar(stat="identity") + 
  xlab("WSI feature") + ylab("# students deeming the feature important (n=216)") + 
  coord_flip() + theme(legend.position = "none") +
  geom_text(label = features$label, y = features$n + 2, size = 4, hjust = 0) +
  geom_hline(yintercept = nrow(d), size = 2, color = "red", alpha = 0.2)
```

### Previously Known WSI Applications

```
wsia = data.frame(app=character(), n=numeric(), stringsAsFactors=FALSE)
wsia[nrow(wsia)+1,] <- list('Mainzer Histo Maps', sum(d$K_MHM))
wsia[nrow(wsia)+1,] <- list('Histoweb - Tübingen', sum(d$K_Histoweb))
wsia[nrow(wsia)+1,] <- list('Histonet - Ulm', sum(d$K_HistonetUlm))
wsia[nrow(wsia)+1,] <- list('HistoWebAtlas - Düsseldorf', sum(d$K_HistoWebAtlas))
wsia[nrow(wsia)+1,] <- list('NUS HISTONET - Marburg', sum(d$K_HistonetMarburg))
wsia[nrow(wsia)+1,] <- list('VMic - Basel', sum(d$K_vMic))
wsia[nrow(wsia)+1,] <- list('Pathorama - Basel', sum(d$K_Pathorama))
wsia[nrow(wsia)+1,] <- list('Virtuelle Pathologie Magdeburg', sum(d$K_virtPatho))
wsia[nrow(wsia)+1,] <- list('Histologiekurs - Zurich', sum(d$K_Histologiekurs))
wsia[nrow(wsia)+1,] <- list('NeoCortex WebMic - Zurich', sum(d$K_NeoCortex))
wsia[nrow(wsia)+1,] <- list('Audiovisueller Kurs in Histopathologie', sum(d$K_AVKurs))
wsia[nrow(wsia)+1,] <- list('Histology - Illinois', sum(d$K_Histology))
wsia[nrow(wsia)+1,] <- list('other', sum(d$K_other))
wsia$label = paste(wsia$n, " (≈", round(wsia$n/nrow(d)*100, 1), "%)", sep = "")

ggplot(wsia, aes(x=reorder(app, n), y=n, color=app, fill=app)) + 
  geom_bar(stat="identity") + 
  geom_hline(yintercept = nrow(d), size = 2, color = "red", alpha = 0.2) +
  xlab("application") + ylab("# students knowing the application (n=216)") + 
  coord_flip() + theme(legend.position = "none") +
  geom_text(label = wsia$label, y = wsia$n + 2, size = 4, hjust=0)
```

### Software Used

#### R

```
##                _                           
## platform       x86_64-redhat-linux-gnu     
## arch           x86_64                      
## os             linux-gnu                   
## system         x86_64, linux-gnu           
## status                                     
## major          3                           
## minor          1.1                         
## year           2014                        
## month          07                          
## day            10                          
## svn rev        66115                       
## language       R                           
## version.string R version 3.1.1 (2014-07-10)
## nickname       Sock it to Me
```

#### Libraries

|  | Package | Version |
| --- | --- | --- |
| acepack | acepack | 1.3-3.3 |
| bitops | bitops | 1.0-6 |
| caTools | caTools | 1.17.1 |
| colorspace | colorspace | 1.2-4 |
| corrplot | corrplot | 0.73 |
| dichromat | dichromat | 2.0-0 |
| digest | digest | 0.6.4 |
| evaluate | evaluate | 0.5.5 |
| formatR | formatR | 1.0 |
| Formula | Formula | 1.1-2 |
| ggplot2 | ggplot2 | 1.0.0 |
| gtable | gtable | 0.1.2 |
| highr | highr | 0.3 |
| Hmisc | Hmisc | 3.14-5 |
| htmltools | htmltools | 0.2.6 |
| knitr | knitr | 1.6 |
| labeling | labeling | 0.2 |
| latticeExtra | latticeExtra | 0.6-26 |
| manipulate | manipulate | 0.98.1062 |
| markdown | markdown | 0.7.4 |
| mime | mime | 0.2 |
| munsell | munsell | 0.4.2 |
| plyr | plyr | 1.8.1 |
| proto | proto | 0.3-10 |
| psych | psych | 1.4.8.11 |
| RColorBrewer | RColorBrewer | 1.0-5 |
| Rcpp | Rcpp | 0.11.2 |
| reshape2 | reshape2 | 1.4 |
| rmarkdown | rmarkdown | 0.3.3 |
| rstudio | rstudio | 0.98.1062 |
| scales | scales | 0.2.4 |
| stringr | stringr | 0.6.2 |
| yaml | yaml | 2.1.13 |
| base | base | 3.1.1 |
| boot | boot | 1.3-11 |
| class | class | 7.3-10 |
| cluster | cluster | 1.15.2 |
| codetools | codetools | 0.2-8 |
| compiler | compiler | 3.1.1 |
| datasets | datasets | 3.1.1 |
| foreign | foreign | 0.8-61 |
| graphics | graphics | 3.1.1 |
| grDevices | grDevices | 3.1.1 |
| grid | grid | 3.1.1 |
| KernSmooth | KernSmooth | 2.23-12 |
| lattice | lattice | 0.20-29 |
| MASS | MASS | 7.3-33 |
| Matrix | Matrix | 1.1-4 |
| methods | methods | 3.1.1 |
| mgcv | mgcv | 1.8-0 |
| nlme | nlme | 3.1-117 |
| nnet | nnet | 7.3-8 |
| parallel | parallel | 3.1.1 |
| rpart | rpart | 4.1-8 |
| spatial | spatial | 7.3-8 |
| splines | splines | 3.1.1 |
| stats | stats | 3.1.1 |
| stats4 | stats4 | 3.1.1 |
| survival | survival | 2.37-7 |
| tcltk | tcltk | 3.1.1 |
| tools | tools | 3.1.1 |
| utils | utils | 3.1.1 |
